# Hyperglycemia causes differential change in macrophage population in the lacrimal gland, conjunctiva and cornea

**DOI:** 10.3389/fimmu.2024.1505508

**Published:** 2024-12-19

**Authors:** Saleh Alfuraih, Amy Tran, Lois Kim, Rais Ansari, Ajay Sharma

**Affiliations:** ^1^ Department of Biomedical and Pharmaceutical Sciences, Chapman University School of Pharmacy, Chapman University, Irvine, CA, United States; ^2^ Department of Pharmaceutical Sciences, Barry and Judy Silverman College of Pharmacy, Health Professions Division, Nova Southeastern University, Fort Lauderdale, FL, United States; ^3^ Department of Pharmacology and Toxicology, Faculty of Pharmacy, Northern Border University, Rafha, Saudi Arabia

**Keywords:** lacrimal gland, conjunctiva, cornea, macrophages, ocular surface, diabetes mellitus, hyperglycemia

## Abstract

**Background:**

Due to its location, the ocular surface is exposed to environmental microbes. Innate immune cells including macrophages are first line defense against infections. *In vitro* exposure to high glucose as well as diabetes-associated hyperglycemia has been shown to affect innate immune cell function and population. The present study was designed to examine the effect of diabetes-associated hyperglycemia on the lacrimal gland, conjunctiva and cornea macrophage population, phenotypic changes and cytokines/chemokines.

**Methods:**

Mouse model of Streptozotocin-induced diabetes was used to induce hyperglycemia. Immunostaining for CD11b and F4/80 was performed to stain macrophages in whole mount cornea, conjunctiva and 50 µm lacrimal gland sections. Flowcytometry was performed on single cell suspension to identify macrophage phenotypes and activation using CD11b, F4/80, CD80, CD206 and MHCII staining. Real time PCR was performed to quantify gene expression for macrophage-associated cytokines (IL-1β, TNF-α, IFN-γ) and chemokine (CCL2).

**Results:**

Our data demonstrates the diabetes-associated hyperglycemia caused a rapid onset and significant decrease in macrophage population in lacrimal gland, conjunctiva and cornea. The onset of this noted decrease was as early as 7 days after hyperglycemia in lacrimal gland and conjunctiva followed by a notable increase towards recovery only in conjunctiva but not in the lacrimal gland. The cornea tissue showed a steady decline up to the tested time point of 28 days. Further, hyperglycemia did not cause any notable changes in macrophage phenotypes, their activation status or the expression of IL-1β, TNF-α, IFN-γ, CCL2 except in the cornea where an increase in the cytokine levels was noted after 7 days of hyperglycemia

**Conclusion:**

Our data shows that diabetes-associated hyperglycemia can cause a significant decrease in microphage population with changing their plasticity or activation status in lacrimal gland, conjunctiva and cornea but the kinetics of decrease and recovery show differential pattern specific for each tissue.

## 1 Introduction

Clinical evidence suggests that patients with diabetes mellitus are at an increased risk of infections (1–3). They also have poor treatment outcomes and increased morbidity (4–7). The incidence of infections is especially high in organs exposed to the environment, including the ocular surface, respiratory tract, genital tract, and skin (5, 6, 8–10). Normal functioning of both innate and acquired immunity is critical to protect against infections. Accumulating evidence demonstrates that diabetes-associated hyperglycemia can compromise both innate and acquired immune responses (11–13). For acquired immunity, studies in diabetic mice models and in patients with diabetes show that hyperglycemia causes a decrease in antibody levels, reduces T- and B-cell proliferation, and impairs germinal center induction (14, 15). Hyperglycemia is animal models and high glucose exposure in cell culture models is also reported to cause lymphopenia (16) reduce lymphocyte viability, induce apoptosis, and increase oxidative stress (14). A decrease in the dendritic cell population in the peripheral blood and pulmonary mucosa has also been demonstrated in diabetic mice and in patients with diabetes (17–19).

Besides acquired immunity, cells involved in the innate immune response are also impacted by diabetes-associated hyperglycemia. For example, multiple studies demonstrate an impairment of neutrophil chemotaxis, migration, ROS production, phagocytosis, and bactericidal activity both in diabetic animal models as well as by *in vitro* high glucose exposure (11, 20, 21). Additionally, hyperglycemia causes impairment of macrophage bactericidal and phagocytic activity, reduces Fcγ receptor expression, and leads to alteration in their plasticity (11, 22–24). It is worthwhile to highlight that the immune impairment in the above-mentioned studies is apparent after acute or short-duration hyperglycemia in contrast to chronic complications of diabetes due to long-duration hyperglycemia. The innate immune response is the first-line defense to protect mucosal surfaces and the associated exocrine glands. The conjunctiva, cornea, lacrimal gland, contains a resident population of innate immune cells, including macrophages (25, 26). However, the effects of diabetes-associated hyperglycemia on the innate immune cells in the lacrimal gland, conjunctiva and cornea unit have not been investigated. Given the evidence that acute or short-duration hyperglycemia can affect the immune cell population and functions, the present study was designed to examine the effect of short-duration hyperglycemia (7, 14, and 28 days) on the lacrimal gland, conjunctiva and cornea macrophages using a mouse model of streptozotocin-induced diabetes mellitus. Since previously published *in vitro* studies show that high glucose exposure can affect bone marrow-derived and peritoneal macrophage phenotypes (11, 22–24, 27), we further tested whether diabetes-associated hyperglycemia can modulate the lacrimal gland, conjunctiva, and cornea macrophage plasticity.

## 2 Materials and methods

### 2.1 Induction of hyperglycemia

The animal experiments were conducted in accordance with the ARVO Statement for the Use of Animals in Ophthalmic and Vision Research. The experiments were approved by the Chapman University Institutional Animal Care and Use Committee. The C57BL/6 strain male mice (Charles River, MA, USA) weighing 25g-30g and ten weeks of age were used. For the induction of diabetes, mice were administered a single intraperitoneal injection of 200 mg/kg streptozotocin dissolved in citrate buffer, pH 4.5. Age-matched male C57BL/6 mice that received an intraperitoneal injection of citrate buffer served as non-diabetic controls. Blood glucose levels were measured using the OneTouch Verio glucometer to confirm the onset of hyperglycemia. The mice were not subjected to any fasting before streptozotocin injection or blood glucose measurement. All animals after the streptozotocin injection had a postprandial blood glucose level of >500 mg/dL. The mice were euthanized to harvest the cornea, conjunctiva, and lacrimal gland at 7, 14, and 28 days after the onset of hyperglycemia.

### 2.2 Immunostaining

The immunostaining for pan macrophage markers, CD11b and F4/80, was performed using full-thickness cornea and conjunctiva. For the lacrimal gland, 50 µM tissue sections were obtained after tissue embedding in optimal cutting temperature (OCT). Briefly, the harvested cornea, conjunctiva, and lacrimal gland were fixed in 4% paraformaldehyde for 75 minutes, followed by five washing with 1X PBS for five minutes each. The fixed tissues were then permeabilized using 0.1% triton X-100 in 1X PBS for 30 minutes at room temperature. The tissues were washed once with 1X PBS for 5 minutes, then blocked with blocking buffer (2% Bovine Serum Albumin, IgG Free in 1X PBS) at room temperature for 30 minutes. The tissues were then incubated in an antibody cocktail containing anti-Mouse CD11b Alexa Fluor-647 (Clone: M1170, BioLegend Inc., San Diego, USA) and F4/80 FITC (Clone: BMB, BioLegend Inc., CA, USA) at 1:25 dilution for each antibody. The tissues were incubated in the antibody cocktail for 18-21 hours overnight on an orbital shaker. The next day, the tissues were washed with 1X PBS five times for ten minutes each and mounted on microscope slides using DAPI-Aqueous Flouro Shield (Abcam, USA). The Z-stack images of the tissue were acquired using a 10X objective lens on the Nikon A1 model confocal microscope (Nikon, NY, USA). Stained cells were quantified using ImageJ software (National Institutes of Health, MD, USA) in a blind manner. The quantification data for immunostaining was obtained by counting twelve images from n=3 mice for each time point.

### 2.3 Gene expression quantification

For gene expression quantification, RNA was isolated from the cornea, conjunctiva, and lacrimal gland using a commercially available kit (RNeasy Mini Kit; Qiagen, CA, USA). The mRNA was immediately reverse-transcribed into cDNA (SuperScript III First Strand; Invitrogen, CA, USA), which was used for quantifying gene expression of macrophage-secreted cytokines and chemokines using primers listed in **Table 1** using real-time PCR. β-actin was used as the housekeeping gene. Briefly, 20 µL reaction mixtures consisting of 2 µL of cDNA, 10 µL of SYBR Master Mix, 2 µL of forward primer, 2 µL of reverse primer, and 4 µL of DEPC water were run at a universal cycle (95°C for 10 minutes, 40 cycles at 95°C for 15 seconds, and 55°C for 60 seconds) using a real-time thermocycler (QuantStudio, Thermo Scientific, IL, USA). Results were normalized to β-actin to calculate ΔCt and fold change in gene expression using the ^ΔΔ^Ct method. The quantification data was obtained from n=3 mice for each time point for each tissue.

**Table 1:** Mouse forward and reverse primers for Real time PCR.

GENE	FORWARD PRIMER	REVERSE PRIMER	NM ACCESSION #
CCL2	GAA GGA ATG GGT CCA GAC ATA C	CAC ATT CAA AGG TGC TGA AGA C	NM_011333.3
IL-1b	CCA CCT CAA TGG ACA GAA TAT CA	CCC AAG GCC ACA GGT ATT T	NM_008361.4
TNF-a	TTG CTC TGT GAA GGG AAT GG	GGC TCT GAG GAG TAG ACA ATA AAG	NM_013693.3
IL-6	TTT CCT CTG GTC TTC TGG AGT A	CTC TGA AGG ACT CTG GCT TTG	NM_031168.2
IL-10	TTG AAT TCC CTG GGT GAG AAG	TCC ACT GCC TTG CTC TTA TTT	NM_010548.2
β-Actin	CTC CCT GGA GAA GAG CTA TGA	CCA AGA AGG AAG GCT GGA AA	NM_007393.5

### 2.4 Flow cytometry quantification

Harvested cornea, conjunctiva, and lacrimal gland were minced into smaller pieces with scissors. The single cell suspension was obtained by incubating the tissue pieces in RPMI media (Gibco, Thermo Scientific, IL, USA) containing 1 mg/mL collagenase D (Millipore Sigma, MA, USA), 0.5 mg/mL DNAse (Invitrogen, CA, USA), and 0.5% BSA on a shaker for 30 minutes at 37°C. The digested samples were filtered through 40 μM cell strainer. The cells were stained with Zombie violet viability dye and CD11b Alexa Fluor 647, F4/80 BV711, CD86 APC/Cy7, CD206 PE, and I-A/I-E PerCP/Cy5.5 (BioLegend Inc., CA, USA) for 30 minutes on ice. The stained cells were analyzed using BD FACSymphony A1 flow cytometer (BD Bioscience, NJ, USA). Results were analyzed using the FlowJo software (TreeStar, Ashland, OR). The quantification data was obtained from n=3 mice for each time point for each tissue.

### 2.5 Bone marrow-derived macrophage culture

To obtain murine macrophage primary culture, hematopoietic stem cells from the bone marrow of femurs and tibias were isolated from C57BL/6 mice. The marrow was flushed out using 1x Ca2+/Mg2+-free PBS with 2% FBS. Red blood cells were lysed by incubating with a commercially available lysis buffer for 3 minutes (BD Biosciences, NJ, USA). The cells were centrifuged at 1500 rpm for 5 minutes and resuspended in 18 ml of Iscove’s Modified Dulbecco’s Medium containing 10% heat-inactivated fetal bovine serum, penicillin/streptomycin and 10 ng/mL m-CSF. The cells were plated in a T75 flask and cultured for a 7-day period to obtain macrophages. The induction of M2 macrophages by carried out by exposing the M0 macrophages to IL-4 (10 ng/ml) for 3 days. To test the effect of a high glucose exposure, the cells were plated in 6-well plate and exposed to media containing 30 mM glucose for 12 and 24 hours. The control cells were cultured in regular media that contained 5 mM of glucose. The experiments were conducted in triplicate.

### 2.6 Effect of high glucose exposure on macrophage apoptosis/necrosis

Twenty-four hours after high glucose exposure, macrophages were trypsinized and collected by centrifugation at 400 x g for 5 minutes. The macrophages were stained using an Annexin V-APC Conjugated Apoptosis Assay Kit (Cayman Chemical, MI, USA). Unstained macrophages were used as the negative control. A 1x Annexin V binding buffer was prepared using a cell-based assay Annexin V binding buffer (10x) at a dilution of 1:10 with DI H20. The cells were resuspended in Annexin V APC/DAPI staining solution and incubated in the dark at room temperature for 10 minutes. The cells were centrifuged at 400 x g for 5 minutes and resuspended in 1x PBS. Necrosis and apoptosis was analyzed using FACS flow cytometry.

### 2.7 Statistical analysis

The data are presented as mean ± standard error of the mean. Statistical analysis was performed using GraphPad Prism software (GraphPad Prism, version 10; GraphPad, San Diego, CA, USA). One-way ANOVA followed by Tukey’s *post-hoc* test was used to analyze the data. Statistical significance was determined as a p value < 0.05.

## 3 Results

### 3.1 Effect of hyperglycemia on macrophages in the lacrimal gland, conjunctiva, and cornea

**Figure 1:**
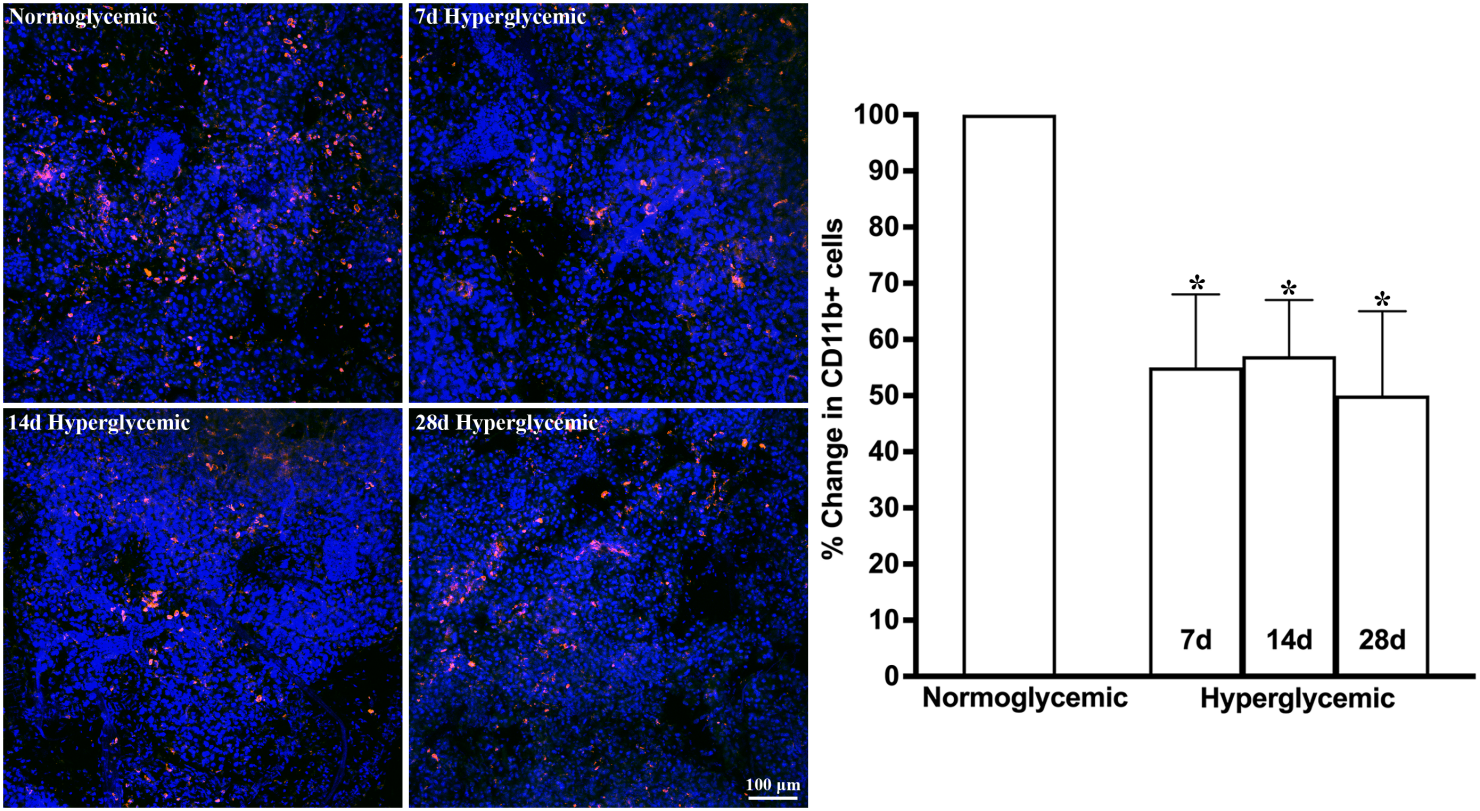
Representative Z-stack confocal images of mouse lacrimal gland showing CD11b immunostaining (red) in normoglycemic nondiabetic mice and diabetic mice with 7, 14, and 28 days (d) of hyperglycemia. Nuclei are stained blue. The graph shows the quantification data of CD11b-stained cells counted in twelve images from n=3 mice for each time point. The Y axis denotes the % change in CD11b-stained cells in hyperglycemic tissues as compared to normoglycemic tissue. * p<0.05 compared to normoglycemic.

**Figure 2:**
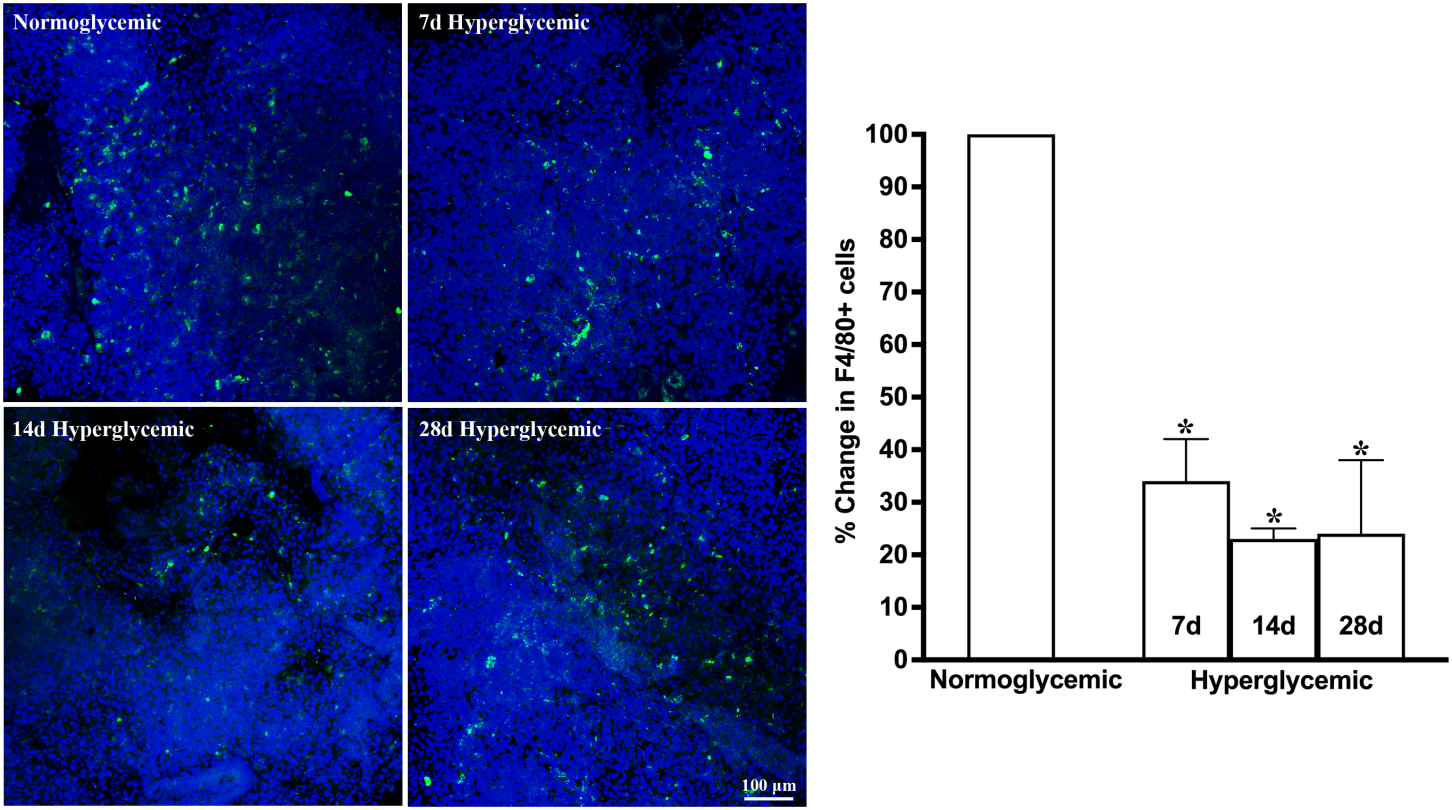
Representative Z-stack confocal images of mouse lacrimal gland showing F4/80 immunostaining (green) in normoglycemic nondiabetic mice and diabetic mice with 7, 14, and 28 days (d) of hyperglycemia. Nuclei are stained blue. The graph shows the quantification data of F4/80-stained cells counted in twelve images from n=3 mice for each time point. The Y axis denotes the % change in F4/80-stained cells in hyperglycemic tissues as compared to normoglycemic tissue. * p<0.05 compared to normoglycemic.

**Figures 1**, **2** show the representative CD11b- and F4/80-stained Z-stack confocal images of the lacrimal gland tissue obtained from non-diabetic normoglycemic mice and diabetic mice after 7, 14, and 28 days of hyperglycemia. As is evident from these images, normoglycemic mice showed CD11b positive (**Figure 1**) and F4/80 positive cells (**Figure 2**), thus demonstrating the presence of macrophages in the healthy lacrimal gland. As can be seen in these images, the 7 and 14 days of hyperglycemia caused a significant decrease in the number of CD11b positive cells and F4/80 cells. Quantification data shown in the graph obtained by counting twelve images each from n=3 mice for each time point revealed an average of 44%, 43%, and 50% decrease in CD11b positive cells in lacrimal gland tissue obtained from diabetic mice with 7, 14, and 28 days of hyperglycemia compared to non-diabetic normoglycemic mice (**Figure 1**). Similar quantification for F4/80 cells revealed a 66%, 77%, and 76% decrease in diabetic mice with 7, 14, and 28 days of hyperglycemia compared to non-diabetic normoglycemic mice (**Figure 2**).

**Figure 3:**
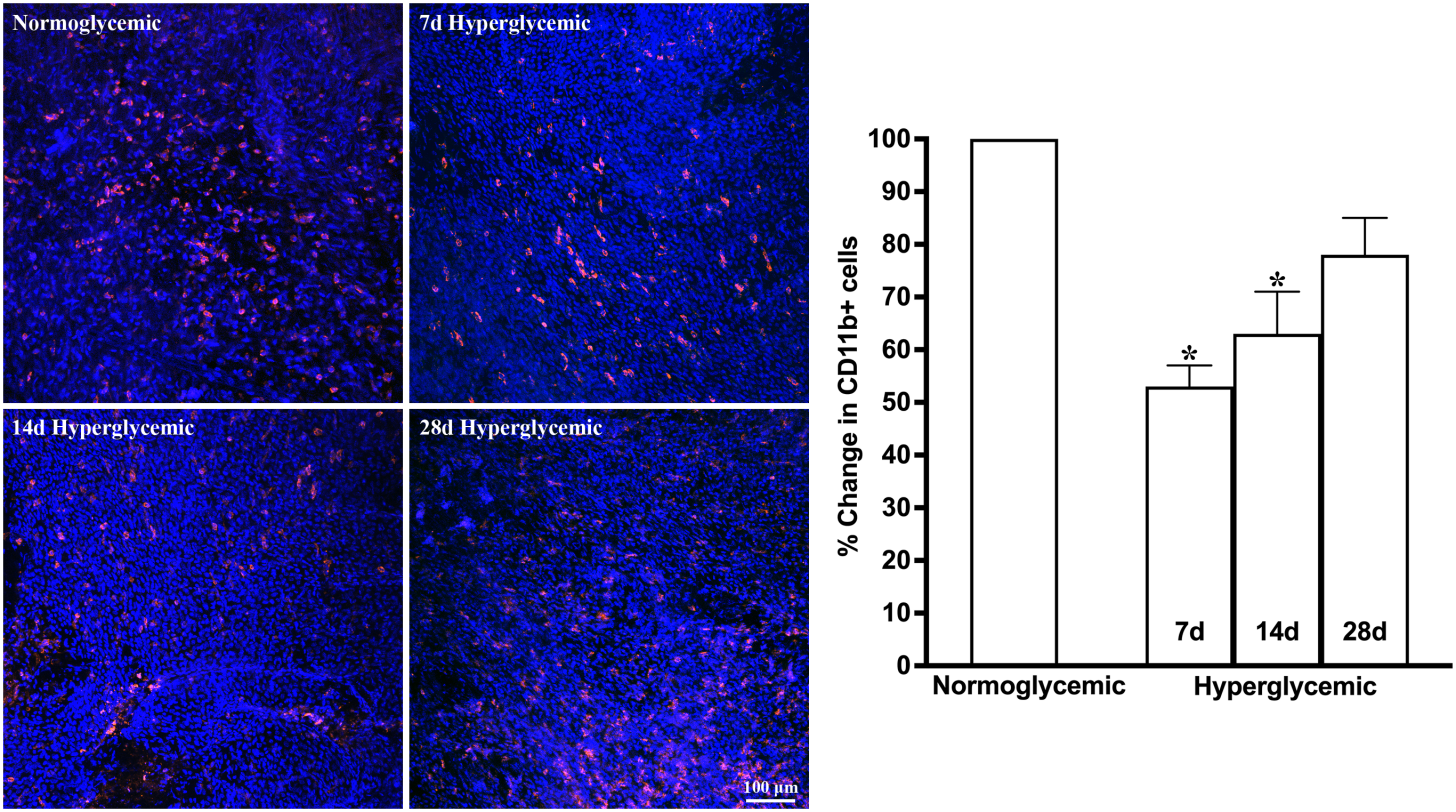
Representative Z-stack confocal images of mouse conjunctiva showing CD11b immunostaining (red) in normoglycemic nondiabetic mice and diabetic mice with 7, 14, and 28 days (d) of hyperglycemia. Nuclei are stained blue. The graph shows the quantification data of CD11b-stained cells counted in twelve images from n=3 mice for each time point. The Y axis denotes the % change in CD11b-stained cells in hyperglycemic tissues as compared to normoglycemic tissue. * p<0.05 compared to normoglycemic.

**Figure 4:**
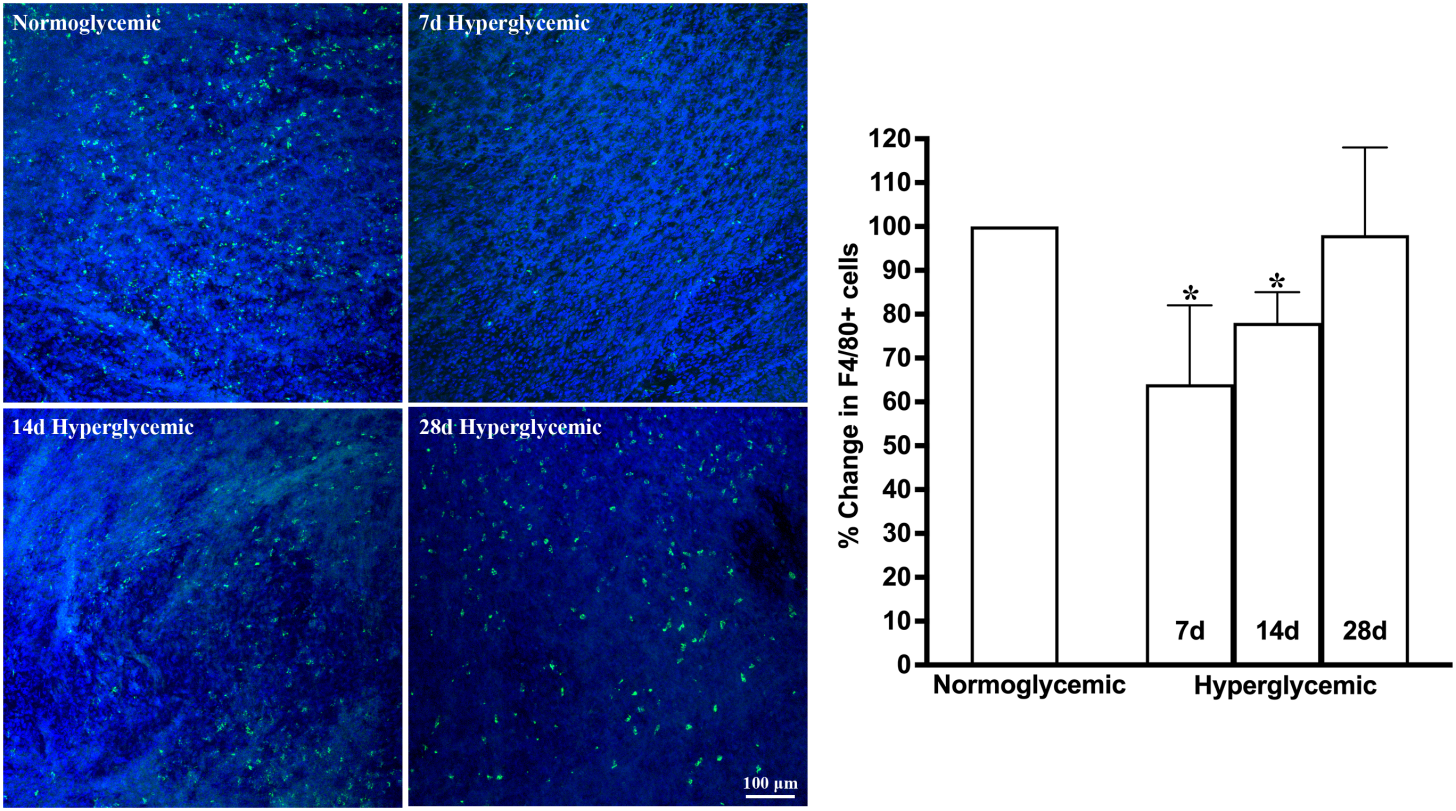
Representative Z-stack confocal images of mouse conjunctiva showing F4/80 immunostaining (green) in normoglycemic nondiabetic mice and diabetic mice with 7, 14, and 28 days (d) of hyperglycemia. Nuclei are stained blue. The graph shows the quantification data of F4/80-stained cells counted in twelve images from n=3 mice for each time point. The Y axis denotes the % change in F4/80-stained cells in hyperglycemic tissues as compared to normoglycemic tissue. * p<0.05 compared to normoglycemic.

**Figures 3**, **4** show the representative Z-stack confocal images of the conjunctiva stained with CD11b and F4/80 obtained from non-diabetic normoglycemic mice and diabetic mice with 7, 14, and 28 days of hyperglycemia. Normoglycemic mice showed CD11b positive (**Figure 3**) and F4/80 positive cells (**Figure 4**), thus confirming the presence of macrophages in the healthy conjunctiva. The 7 and 14 days of hyperglycemia caused a significant decrease in the number of CD11b positive cells and F4/80 cells in the conjunctiva, similar to the one noted in the lacrimal gland. However, unlike the lacrimal gland, the number of CD11b and F4/80-stained cells started to recover back at 28 days of hyperglycemia. Quantification data shown in the graph revealed an average of 47%, 37%, and 21% decrease in CD11b positive cells (**Figure 3**) and a 35%, 22%, and 5% decrease in F4/80 cells compared to normoglycemic mice (**Figure 4**).

**Figure 5:**
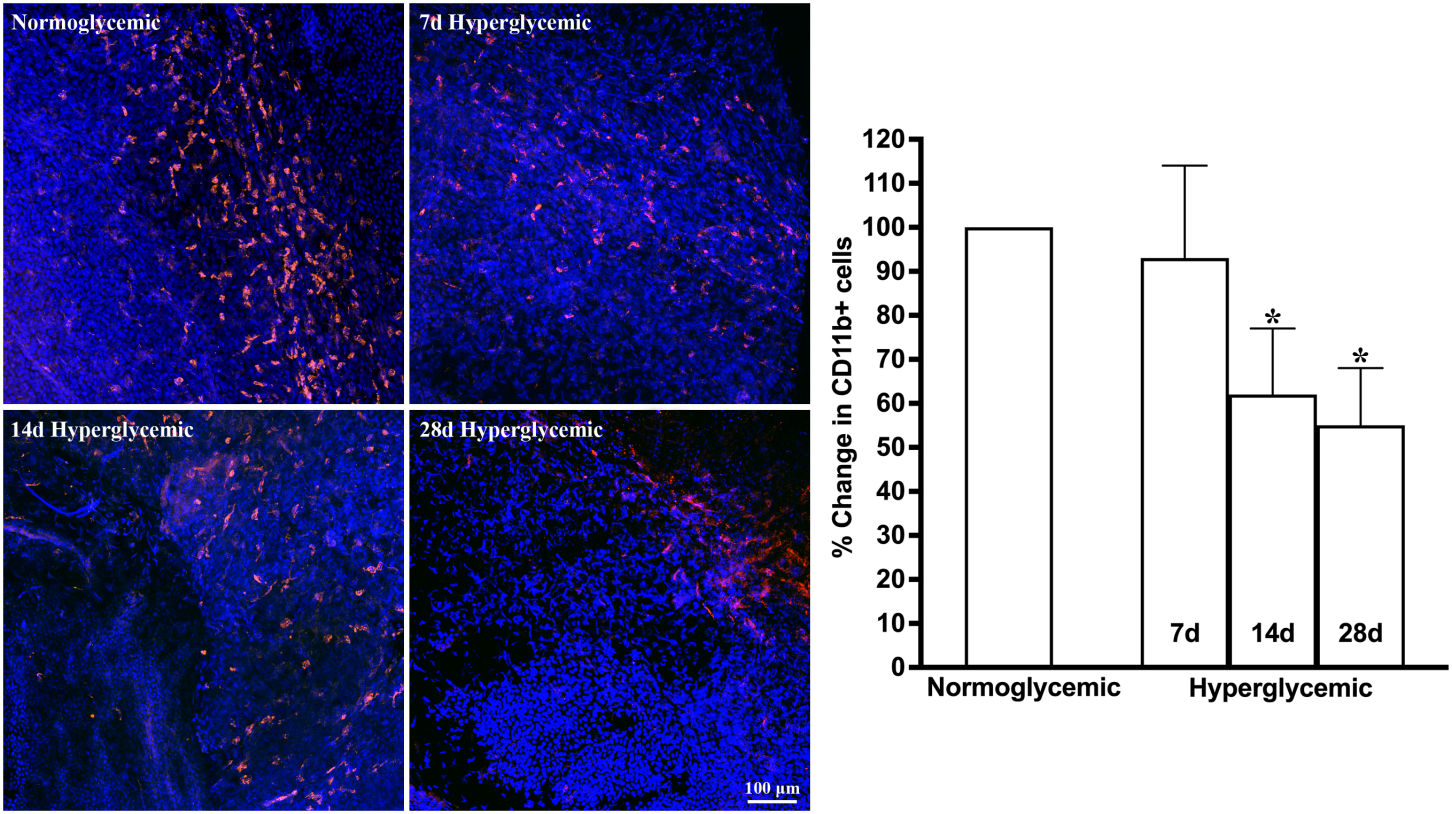
Representative Z-stack confocal images of mouse cornea showing CD11b immunostaining (red) in normoglycemic nondiabetic mice and diabetic mice with 7, 14, and 28 days (d) of hyperglycemia. Nuclei are stained blue. The graph shows the quantification data of CD11b-stained cells counted in twelve images from n=3 mice for each time point. The Y axis denotes the % change in CD11b-stained cells in hyperglycemic tissues as compared to normoglycemic tissue. * p<0.05 compared to normoglycemic.

**Figure 6:**
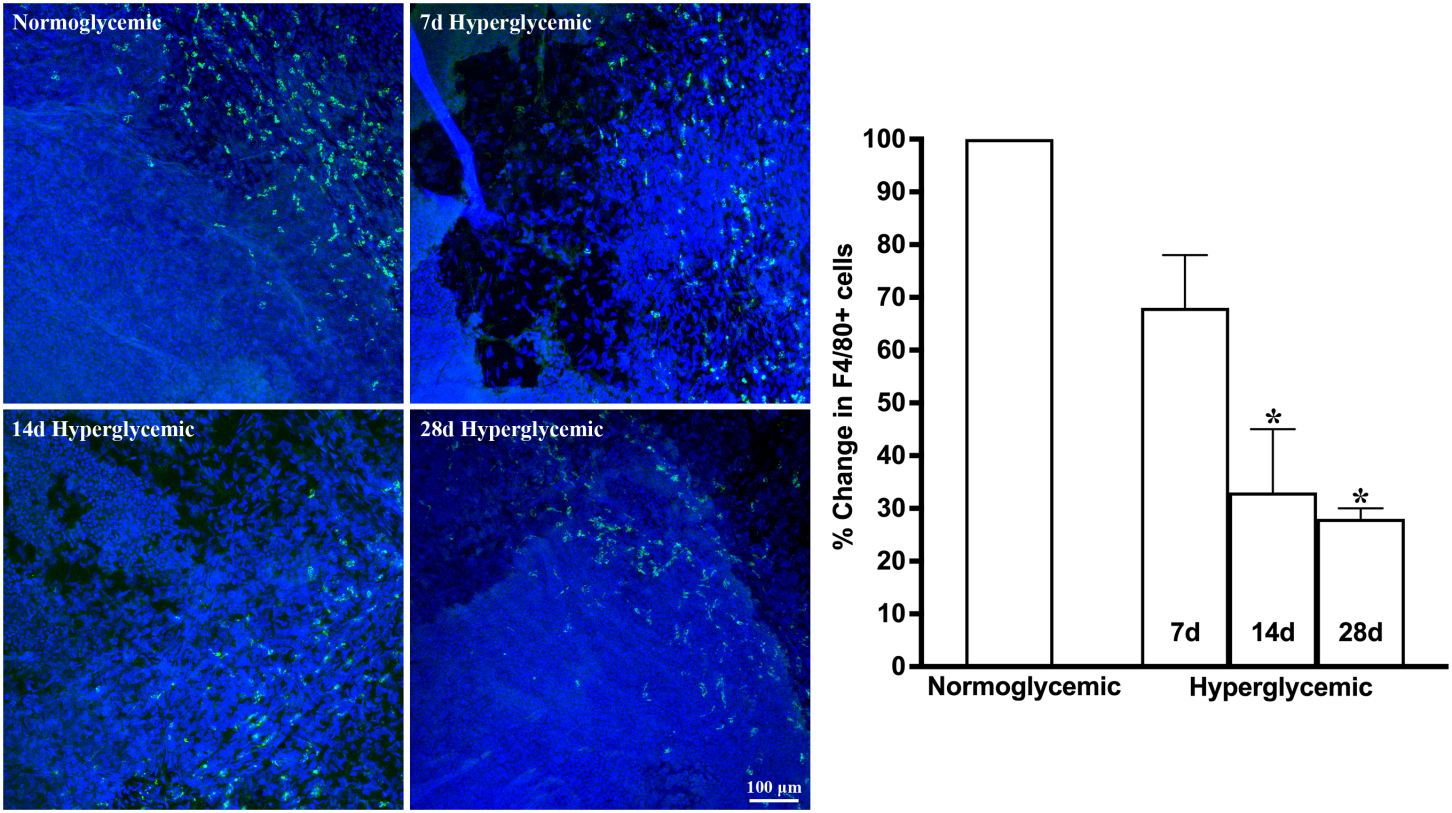
Representative Z-stack confocal images of mouse cornea showing F4/80 immunostaining (green) in normoglycemic nondiabetic mice and diabetic mice with 7, 14, and 28 days (d) of hyperglycemia. Nuclei are stained blue. The graph shows the quantification data of F4/80-stained cells counted in twelve images from n=3 mice for each time point. The Y axis denotes the % change in F4/80-stained cells in hyperglycemic tissues as compared to normoglycemic tissue. * p<0.05 compared to normoglycemic.

**Figures 5**, **6** show the representative Z-stack confocal images of the cornea stained with CD11b and F4/80 obtained from non-diabetic normoglycemic mice and diabetic mice with 7, 14, and 28 days of hyperglycemia. The CD11b and F4/80 cells were mostly localized in the periphery with a few micrometers from the limbus. In contrast to the lacrimal gland and conjunctiva, where the most notable decrease was observed at 7 days of hyperglycemia, in the cornea, this decrease was most notable at 28 days, followed by 14 days and a very minimal decrease observed at 7 days of hyperglycemia. Quantification data revealed a 6%, 37%, and 44% decrease in CD11b-stained cells and a 31%, 66%, and 72% decrease in F4/80-stained cells at 7, 14, and 28 days of hyperglycemia as compared to normoglycemic mice.

### 3.2 Effect of hyperglycemia on macrophage subtypes and MHCII activation

**Figure 7:**
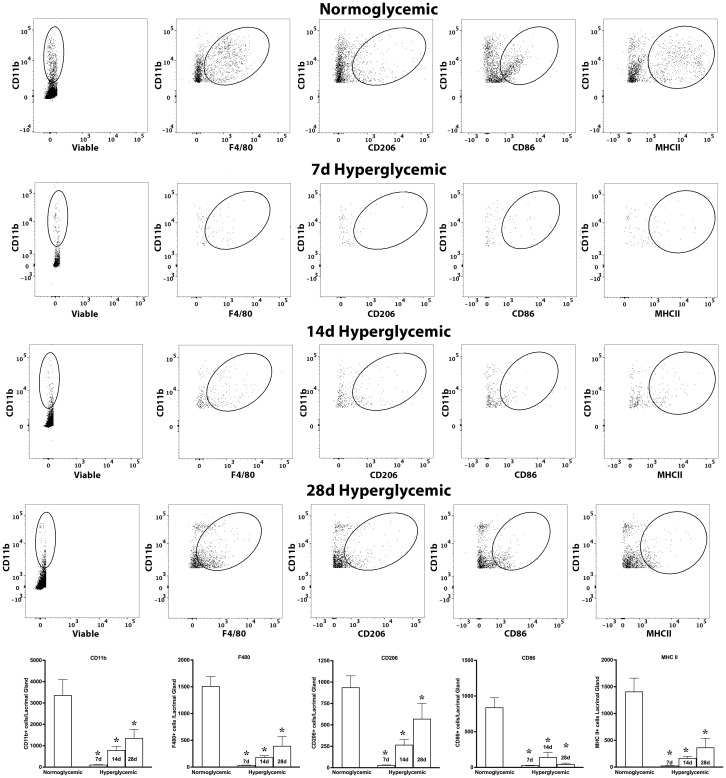
Representative flow cytometric plots showing different macrophage populations and MHC II expression in the lacrimal gland cell suspension obtained from normoglycemic nondiabetic mice and diabetic mice with 7, 14, and 28 days (d) of hyperglycemia. The graph shows quantitative data obtained from the lacrimal gland of n=3 mice for each time point. * p<0.05 compared to normoglycemic.

Next, we tested whether hyperglycemia causes any change in macrophage phenotype and activation status. To test this, we quantified the macrophage subtypes using flow cytometry after staining the cell suspensions for macrophage markers for M0 (F4/80), proinflammatory (CD86), and regulatory (CD206) macrophage phenotypes and also their activation status using MHCII. **Figure 7** shows representative flow cytometry panels and quantification plots of lacrimal gland cell suspensions obtained from normoglycemic non-diabetic mice and diabetic mice after 7,14, and 28 days of hyperglycemia. As can be seen from the flowcytometry data, hyperglycemia caused a rapid and robust decrease in the number of CD11b, CD11b+F4/80, CD11b+CD206, CD11b+CD86, and CD11b+MHCII double-positive cells in the lacrimal gland. This decrease was noted as early as 7 days of hyperglycemia, and the number of cells remained significantly low up to the tested duration of 28 days.

**Figure 8:**
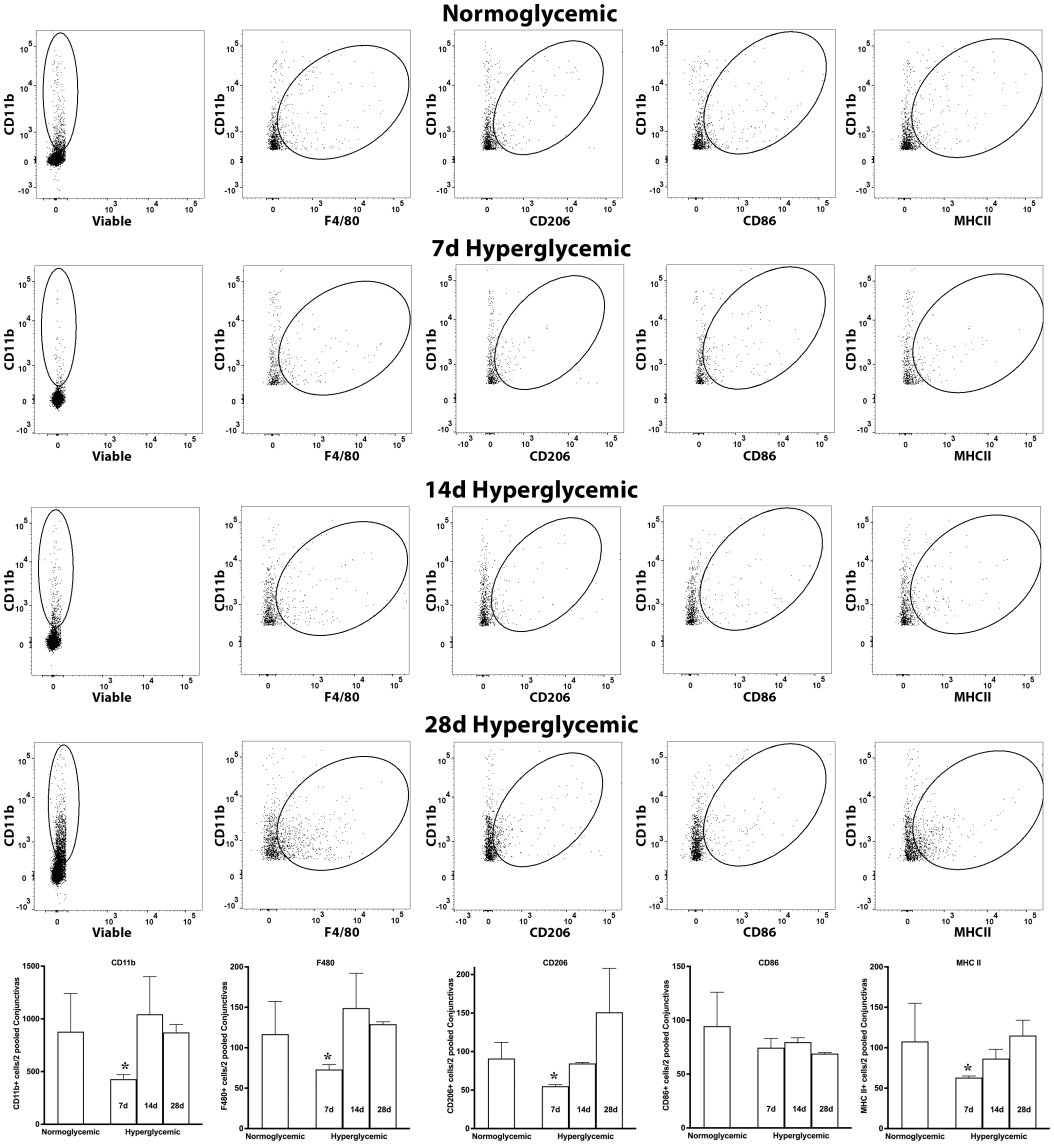
Representative flow cytometric plots showing different macrophage populations and MHC II expression in the conjunctiva cell suspension obtained from normoglycemic nondiabetic mice and diabetic mice with 7, 14, and 28 days (d) of hyperglycemia. The graph shows quantitative data obtained from the two pooled conjunctivas and n=3 mice for each time point. * p<0.05 compared to normoglycemic.

**Figure 8** shows representative flow cytometry panels and quantification plots of conjunctiva cell suspensions obtained from normoglycemic non-diabetic mice and diabetic mice after 7,14, and 28 days of hyperglycemia. Like the lacrimal gland, hyperglycemia caused a significant decrease in the number of CD11b, CD11b+F4/80, CD11b+CD206, and CD11b+MHCII double-positive cells on day 7. However, unlike the lacrimal gland there was a significant recovery of these macrophage populations at day 14 and day 28 of hyperglycemia. Furthermore, hyperglycemia did not modulate the number of CD11b+CD86 double-positive proinflammatory macrophages in the conjunctiva.

**Figure 9:**
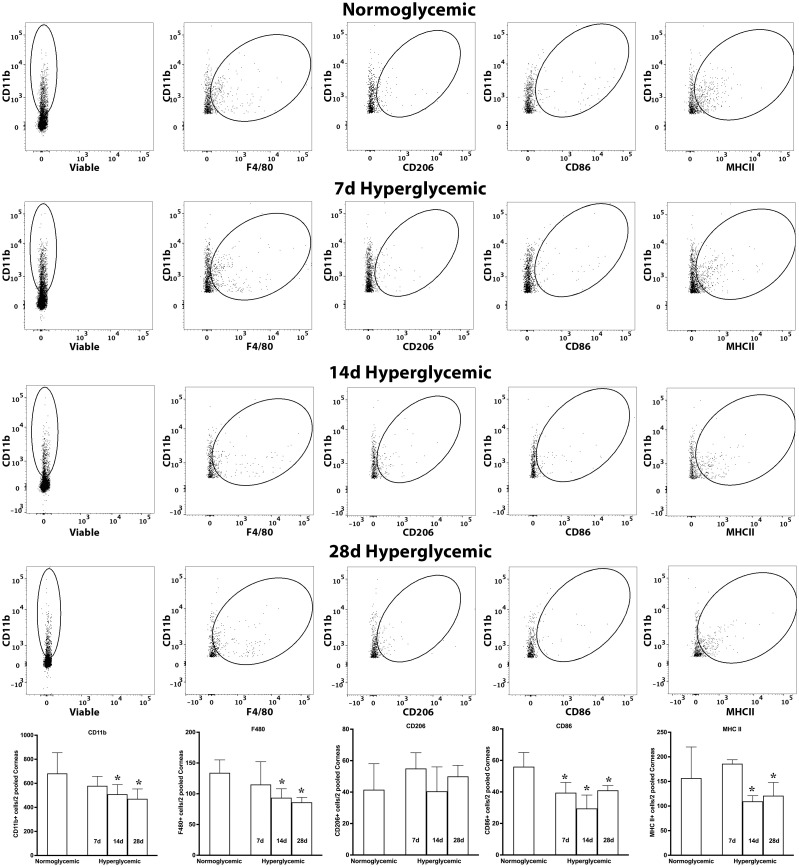
Representative flow cytometric plots showing different macrophage populations and MHC II expression in the cornea cell suspension obtained from normoglycemic nondiabetic mice and diabetic mice with 7, 14, and 28 days (d) of hyperglycemia. The graph shows quantitative data obtained from the two pooled corneas and n=3 mice for each time point. * p<0.05 compared to normoglycemic.

**Figure 9** shows representative flow cytometry panels and quantification plots of corneal cell suspensions obtained from normoglycemic non-diabetic mice and diabetic mice after 7,14, and 28 days of hyperglycemia. Hyperglycemia caused a significant decrease in CD11b, CD11b+F4/80, and CD11b+MHCII double-positive cells only at 14 and 28 days after hyperglycemia. Moreover, no significant decrease in CD11b+CD206 double-positive cells was noted, whereas a reduction in CD11b+CD86 double-positive cells was observed at all the 3 tested time points after hyperglycemia.

### 3.3 Effect of hyperglycemia on macrophage-secreted cytokine and chemokine gene expression

**Figure 10:**
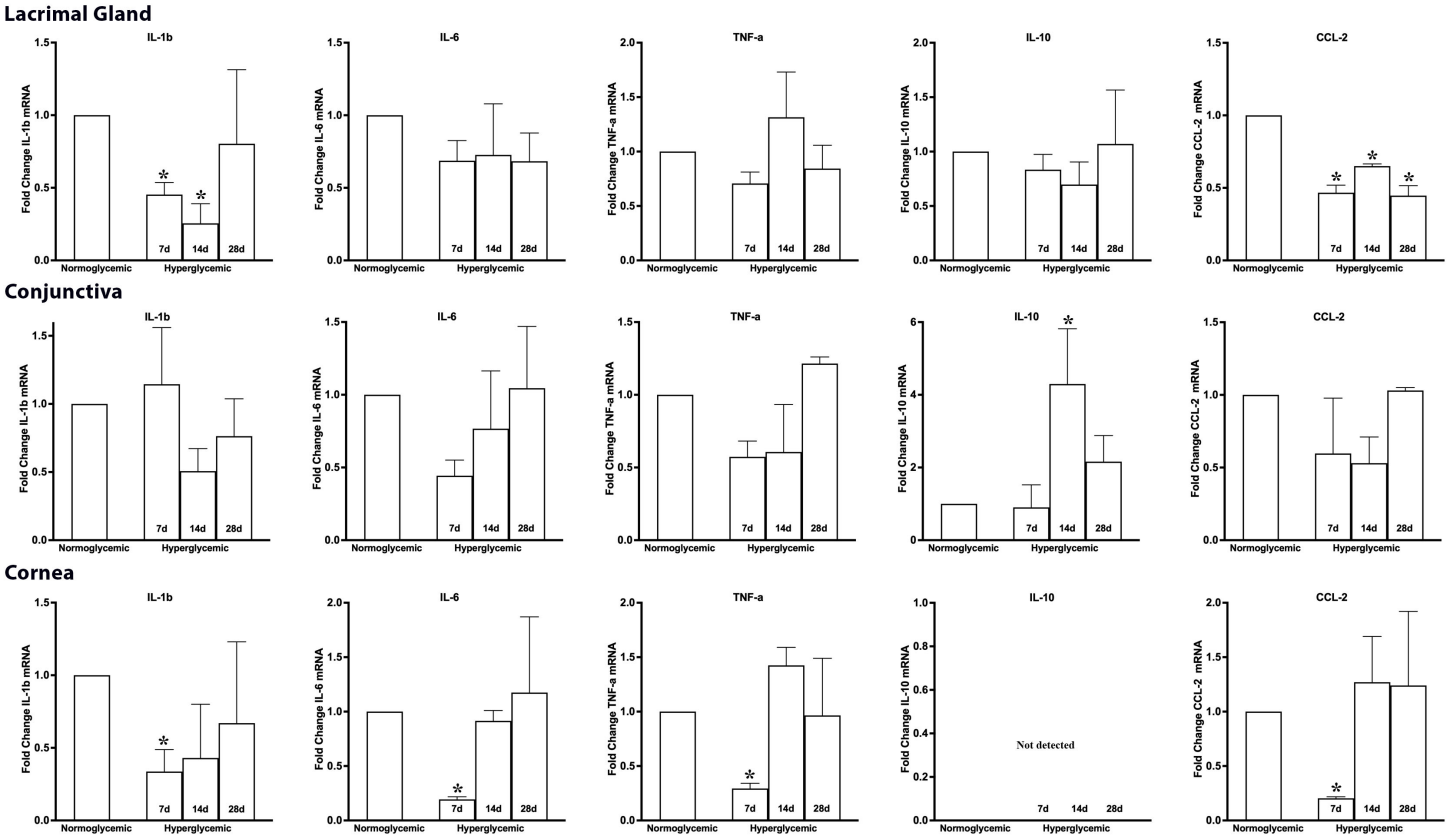
Fold change in the gene expression of macrophage-secreted cytokines and chemokines in lacrimal gland, conjunctiva and corneal lysates obtained from normoglycemic nondiabetic mice and diabetic mice with 7, 14, and 28 days (d) of hyperglycemia. * p<0.05 compared to normoglycemic.

Next, we examined whether hyperglycemia could modulate the gene expression of macrophage-secreted cytokines and chemokines. To test this hypothesis, we quantified the gene expression of macrophage-secreted cytokines (IL-1β, IL-6, TNF-α, IL-10) and chemokines (CCL2) in the lacrimal gland, conjunctiva and cornea of non-diabetic normoglycemic mice and diabetic mice with 7, 14, and 28 days of hyperglycemia. **Figure 10** (top panel) shows that 7,14, or 28 days of hyperglycemia did not cause any significant changes in the gene expression of IL-6, TNF-α, or IL-10 in the lacrimal gland, but a significant decrease in the expression of IL-1β, and CCL2 was observed. **Figure 10** (middle) panel shows that hyperglycemia did not cause any significant changes in the gene expression of IL-1β, IL-6, TNF-α, and CCL2 in the conjunctiva, but a significant increase in IL-10 expression was noted at 14 days of hyperglycemia. Lastly, a significant decrease in the expression of IL-6, TNF-α, and CCL2 was noted in the cornea at 7 days after hyperglycemia, whereas no significant alterations in the expression of these genes could be noted at 14 or 28 days of hyperglycemia. Furthermore, IL-10 expression was not detected either in euglycemic or hyperglycemic cornea. No TGF-β 1 levels were detected and SMA staining did not reveal any fibrosis in tissues obtained from diabetic mice (Data not shown).

### 3.4 Effect of high glucose exposure on macrophage viability

**Figure 11:**
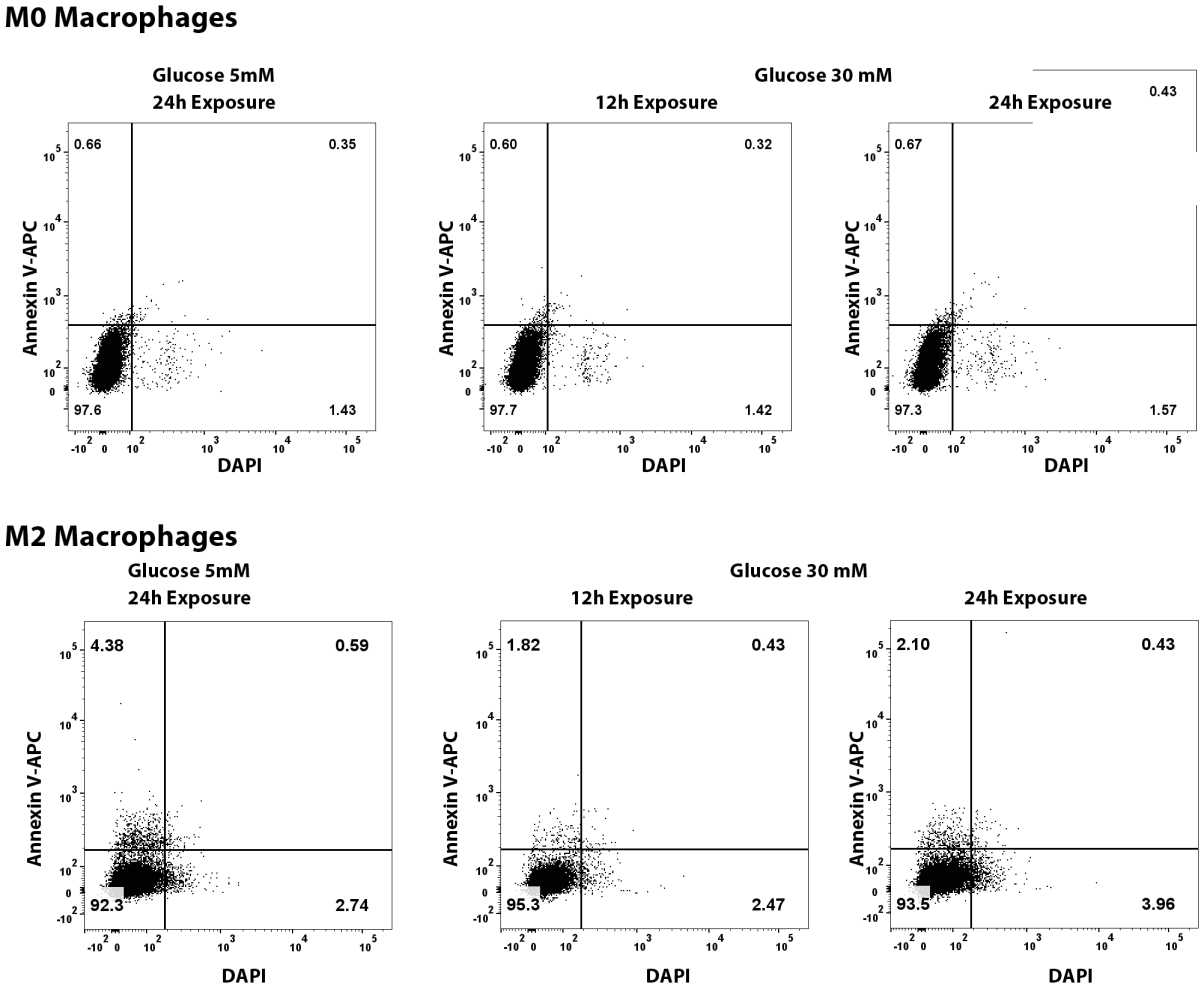
Flowcytometry quantification of apoptosis and necrosis of M0 and M2 macrophages exposed to normal (5mM glucose) or high glucose (30 mM) for 12 and 24 hours. The cells were stained with Annexin V-APC and DAPI using a commercially available kit.

To test whether the noted decrease in macrophage population in the diabetic mice is due to direct toxic effect of high glucose-associated osmotic stress, we exposed the primary cultures of murine M0 and M2 macrophages to high glucose (30mM) for 12 and 24 hours and quantified the apoptotic and necrotic cell death by flowcytometry after Annexin V and propidium iodide (PI) staining. The flowcytometry data shows that high glucose exposure caused neither any significant decrease in macrophage viability, nor any increase in apoptosis or necrosis compared to the cells exposed to normal 5mM glucose (**Figure 11**).

## 4 Discussion

The lacrimal functional unit components lacrimal gland, conjunctiva, and cornea contains a resident population of innate immune cells (25, 26), thus offering an opportunity to test the effects of hyperglycemia on macrophages in these tissues. The results of our study for the first time demonstrate that short-duration hyperglycemia causes a decrease in the macrophage population in the lacrimal gland, conjunctiva, and cornea. These rapid-onset effects of hyperglycemia can be especially relevant for patients with poor glycemic control experiencing short-duration hyperglycemia. Our results are in agreement with previous publications showing that acute and short-duration high glucose levels can cause a decrease in immune cells in peripheral blood, lung mucosa, and spleen (16–19). It is interesting to note that the onset, magnitude, and recovery of this reduction showed a differential pattern between the three tissues. In the lacrimal gland, the decrease was rapid, and the levels remained low up to the tested time point of 28 days. On the other hand, this decrease in the cornea was more gradual and showed a time-dependent decline, with the lowest numbers noted at 28 days of hyperglycemia. Finally, the conjunctiva, like the lacrimal gland, showed a rapid onset decrease, followed by a recovery back towards a higher number of cells. This difference in immune cell recovery between conjunctiva and lacrimal can be either due to differences in recruitment from peripheral blood since hyperglycemia is also known to impair immune cell recruitment (11–13). Therefore, it may be possible that adhesion molecules and other factors critical for cell recruitment may be impacted to a greater extent in the lacrimal gland compared to the conjunctiva. Alternatively, the small thickness and high vascularity of conjunctiva may allow for a faster and more efficient replenishment of the immune cells compared to lacrimal gland.

*In vitro* studies demonstrate that high glucose exposure of bone marrow-derived and peritoneal macrophages can alter their phenotype to the M2 subtype (22, 27). Therefore, we next tested whether short-duration hyperglycemia can cause an increase in proinflammatory or a decrease in the regulatory phenotype subpopulations of macrophages in the lacrimal gland, conjunctiva and cornea. In contrast to the *in vitro* studies, our data shows that hyperglycemia does not cause any major phenotypic changes in the macrophages in any of the three tissues since no increase in proinflammatory or regulatory macrophage populations was observed. On the contrary, hyperglycemia caused a significant decrease in both proinflammatory and regulatory macrophage subtypes in the lacrimal gland and a significant decrease in the regulatory subtype in the conjunctiva. In the cornea, a significant decrease in the inflammatory subtype macrophages was observed. Interestingly, the cornea showed an increased gene expression of macrophage-derived proinflammatory cytokines, IL-1β, TNF-α, IL-6, and chemokine CCL2, which could likely be a compensatory response to the declining number of proinflammatory macrophages. Furthermore, a significant decrease in the number of activated macrophages was also noted in all three tissues.

The ocular surface, comprised of cornea and conjunctiva, is in direct contact with environmental microbes, making it prone to infection. Previous data from our lab and others have shown that hyperglycemia causes a decrease in corneal and conjunctival epithelial barrier function, and a compromised barrier function increases the risk of microbial penetration into the ocular surface (28–31) Innate immune cells are the first-line defense against infections. The hyperglycemia-mediated decrease in the corneal and conjunctival macrophages noted in the present study may further increase the risk of ocular surface infections along with impaired epithelial barrier function. Indeed, several studies demonstrate an increased incidence of corneal and conjunctival infections in patients with poor glycemic control (10, 32, 33). Contrary to the cornea and conjunctiva, the lacrimal gland does not have a surface location. In addition to fighting microbial infections, macrophages, also participate in tissue repair and phagocytosis of senescent cells (34). Macrophages also play a crucial role in lymph angiogenesis and neuronal proliferation (35, 36). A robust decrease noted in the macrophage population in the lacrimal gland raises the possibility that poor glycemic control can compromise these macrophage-mediated vital functions in the lacrimal gland.

In summary, our data shows that hyperglycemia can cause a decrease in the macrophage population in the lacrimal gland, conjunctiva and cornea, which, in turn, can compromise the vital functions mediated by these immune cells, thus affecting the health of the lacrimal functional unit in diabetes mellitus. Therefore, besides long-term glycemic control, management of short-duration hyperglycemia may also be important for preventing diabetes-associated ocular surface damage and the risk of infections.

## Data Availability

The original contributions presented in the study are included in the article/**Supplementary Material**. Further inquiries can be directed to the corresponding author.
