# Supplementary figures and images for: Hyperglycemia causes differential change in macrophage population in the lacrimal gland, conjunctiva and cornea
Source: Front Immunol. 2024 Dec 19;15:1505508. doi: 10.3389/fimmu.2024.1505508 (PMC11693596; doi:10.3389/fimmu.2024.1505508)

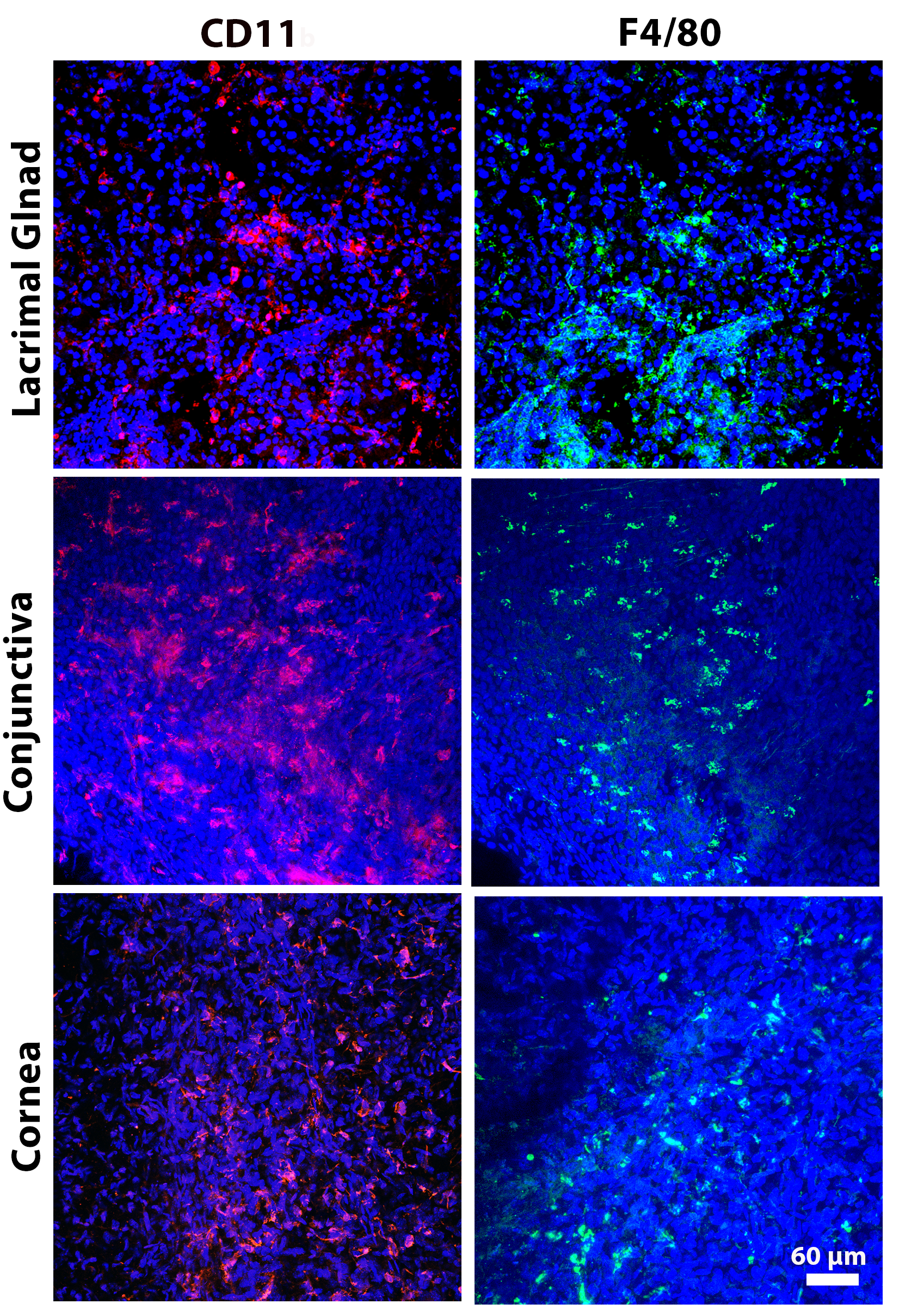

Supplement: Supplementary file 1 [file Image1.tif]
